# Presentation of COVID-19 infection with bizarre behavior and encephalopathy: a case report

**DOI:** 10.1186/s13256-021-02851-0

**Published:** 2021-04-28

**Authors:** Zahra Teimouri-Jervekani, Mehrzad Salmasi

**Affiliations:** 1 Cardiac Rehabilitation Center, Cardiovascular Research Institute, Isfahan University of Medical Sciences, Isfahan, Iran; 2 Internal Medicine Department, Isfahan University of Medical Sciences, Isfahan, Iran

**Keywords:** COVID-19, Encephalopathy, Bizarre behavior

## Abstract

**Background:**

Current studies show that patients with severe coronavirus disease 2019 (COVID-19) have neurological symptoms manifesting as acute cerebrovascular diseases, impaired consciousness, and skeletal muscle symptoms. Bizarre behavior is an unusual and unique presenting symptom of COVID-19 infection in our patient.

**Case presentation:**

We report a case of COVID-19 infection in a middle aged Iranian man without underlying disease who presented with bizarre behavior. Results of brain imaging were normal, but COVID-19 pneumonia was detected on chest computed tomography scan. Given the respiratory problem and positive polymerase chain reaction (PCR) test for COVID-19, treatment with hydroxychloroquine was administered, and after 2 days all of the symptoms resolved.

**Conclusions:**

Encephalopathy and encephalitis may be a possible presentation of COVID-19. Clinicians and health care providers should consider the presence of COVID-19 with bizarre behavior during this COVID-19 pandemic.

## Background

In December 2019, Wuhan, in Hubei province, China, became the center of an outbreak of coronavirus disease 2019 (COVID-19)-induced pneumonia [1]. This disease was diagnosed for the first time in Iran in February 2020, and the coronavirus outbreak then increased rapidly [2].

COVID-19 infection has a wide spectrum of clinical symptoms including asymptomatic infection, mild and severe forms of respiratory problems, and even death due to respiratory failure [3].

Current studies show that patients with severe COVID-19 have neurological symptoms manifesting as acute cerebrovascular diseases, impaired consciousness, and skeletal muscle symptoms [4]. Altered mental status can be the presenting symptom of COVID-19 infection in older patients. One case of encephalopathy was reported in a 72-year-old man with underlying disease who was infected with COVID-19 [5].

Here we report a case of COVID-19 infection in a middle-aged man who presented with encephalopathy. This study was approved by the ethics committee of Isfahan University of Medical Sciences (IR.MUI.MED.REC.1399.913).

## Case presentation

A 53 year old Iranian man without any past medical history presented to the emergency department with symptoms of severe headache and bizarre behavior. Symptom onset occurred 2 weeks before admission; the first symptoms were fever and myalgia. The fever lasted 3 days, and then cough and dyspnea appeared. With the progression of dyspnea, the patient experienced some episodes of severe generalized headache. The headache became progressively worse, and on the day of his admission, he exhibited bizarre behavior. For example, he undressed, removing his shirt and trousers, in front of his family and also tried to urinate in the room. Due to the appearance of dysarthria that morning and other problems, he was admitted to the emergency department. He had no specific family or social history. On physical exam, except for dysarthria, he had normal vital signs, no sign of hypoxia, and normal neurological exam. The patient was alert and oriented to time, place, and person. No neck stiffness, Kernig’s sign, or Brudzinski’s sign was detected.

**Fig. 1:**
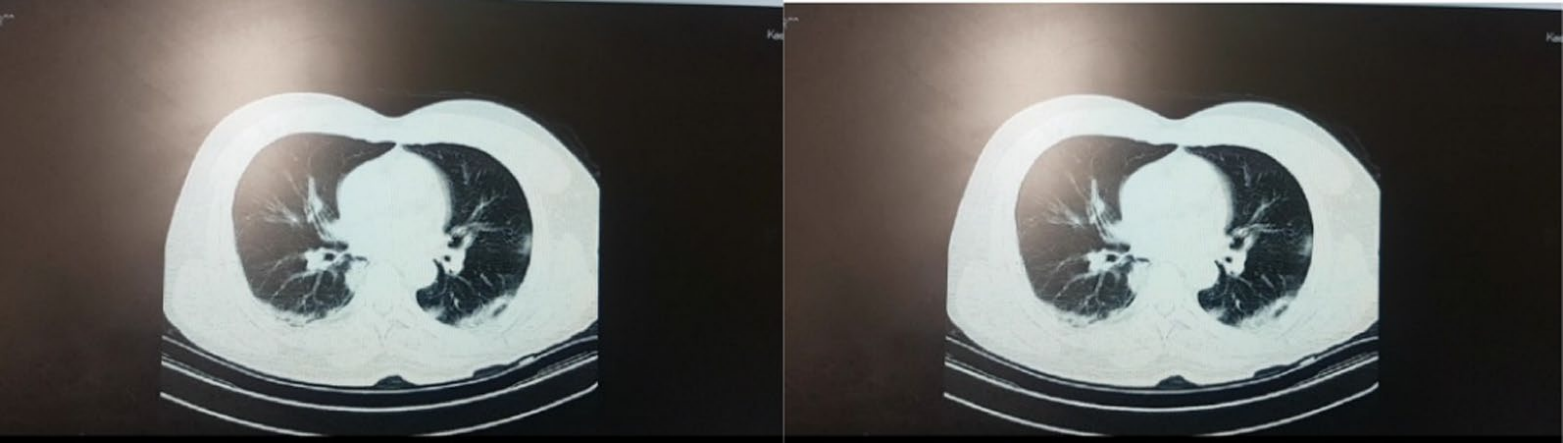
Lung high-resolution computed tomography

Routine laboratory tests and chest and brain computed tomography (CT) were performed. Laboratory test results are summarized in Table 1. Chest CT revealed bilateral peripheral ground-glass opacities suggestive of COVID-19 infection (Fig. 1).

**Table 1:** Laboratory tests

White blood cells/µL	8700	4000–10000
Hemoglobin g/dL	14.6	14–18
Platelets/µL	353 × 10^3^	150–450 × 10^3^
Lymphocytes %	11.1	
Neutrophils %	83	
Erythrocyte sedimentation rate mm/hour	67	0–12
C-reactive protein mg/L	12	0–6
Blood urea nitrogen mg/dL	24	8.4–25.7
Creatinine mg/dL	0.81	0.7–1.4
Sodium mEq/L	144	135–145
Potassium mEq/L	4	3.8–5
Magnesium mEq/L	2.2	1.8–2.6
Calcium mg/dL	8.56	8.5–11
Phosphorus mg/dL	2.88	2.6–4.5
Albumin g/dL	4.2	3.5–5.2
Aspartate aminotransferase U/L	27	0–38
Alanine aminotransferase U/L	16	0–41
Alkaline phosphatase U/L	124	80–306
pH	7.33	
PCO_2_ mmHg	37.2	
HCO_3_ mEq/L	19.6	
Creatine phosphokinase IU/L	100	21–232
Glucose mg/dL	96	70–115
COVID-19 RT-PCR	Positive	
Blood culture	Negative	
Toxin panel	Negative for opioids, amphetamine and methamphetamine, tricyclic antidepressants, benzodiazepine	
Urinalysis	Normal	

*PCO*_*2*_ partial pressure of carbon dioxide, *HCO*_*3*_ bicarbonate, *RT-PCR* reverse transcription polymerase chain reaction

**Fig. 2:**
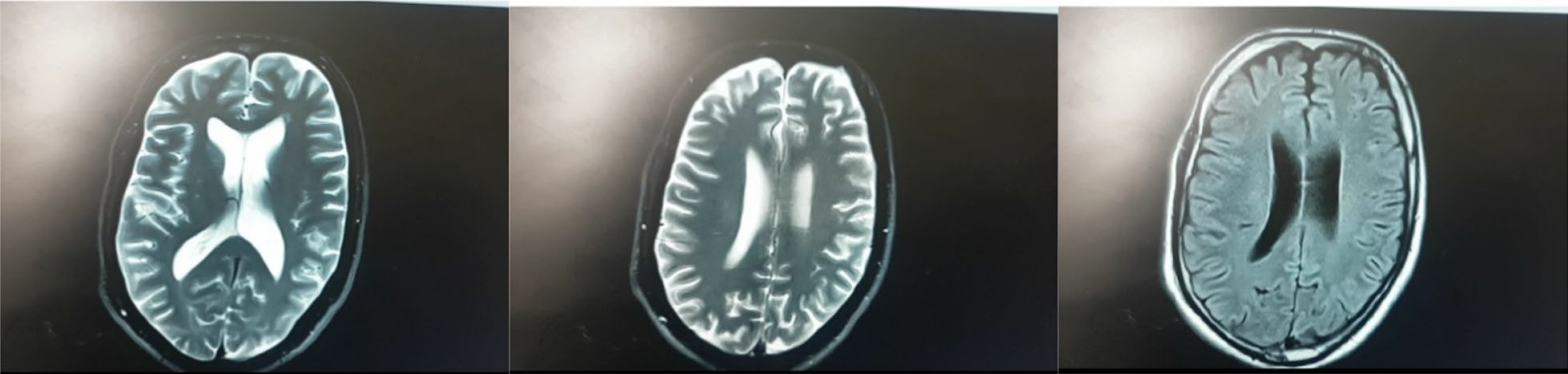
Brain magnetic resonance imaging with diffusion-weighted imaging

Due to normal brain CT results, brain magnetic resonance imaging (MRI) was performed to evaluate the cause of dysarthria and bizarre behavior (Fig. 2). The brain MRI results were normal.

Based on the respiratory problem and positive polymerase chain reaction (PCR) test for COVID-19, the patient was treated with hydroxychloroquine (200 mg twice daily for 5 days). After 2 days, dysarthria and abnormal behavior were resolved completely. The patient was discharged on day 4, with resolution of respiratory and neurological signs and symptoms.

## Discussion

The signs and symptoms of COVID-19 present at illness onset vary from mild or nonspecific symptoms to severe respiratory distress, and non-respiratory symptoms have also been reported [6]. Common neurological manifestations reported for COVID-19 are acute stroke, impaired consciousness, and muscle injury. Patients with severe cases and older patients are more susceptible to these complications [4]. In light of the lack of sufficient data on COVID-19, a review of past experience with neurological aspects of previous forms of coronavirus was carried out. Although the data are sparse, rare cases of acute disseminated encephalomyelitis (ADEM)-like demyelination, encephalitis, and brainstem encephalitis have been reported for Middle East respiratory syndrome (MERS), severe acute respiratory syndrome (SARS), and other types [7–10]. One report described a case of encephalopathy in a 72-year-old man with underlying neurological disease who was infected with COVID-19 [4]. The severe acute respiratory syndrome coronavirus 2 (SARS-CoV-2) invades the brain by various routes, such as binding to the ACE2 receptor on neurons and endothelial cells, through the olfactory system and spread across the cribriform plate, and by crossing the blood–brain barrier via infected leukocyte migration by a Trojan horse mechanism. Encephalopathy is reported in older patients and patients with severe or critical disease [11–13]. Our patient was middle-aged, without any previous medical history, and with normal brain imaging and non-severe infection. Due to the relatively rapid resolution of neurological symptoms and no pathological findings in the imaging, further studies were not performed. The etiology of encephalopathy or possible encephalitis in COVID-19 or other coronaviruses remains poorly understood, and could be due to misdirected host immune responses [14]. Neurological manifestations of COVID-19 derive from both direct invasion and indirect effects due to hyperinflammation and encephalopathy [15]. As the number of patients with COVID-19 increases worldwide, clinicians should be watchful for patients presenting with bizarre behavior or altered mental status. Possible spread from the respiratory tract to the central nervous system must be considered, and cerebrospinal fluid analysis for virus detection is recommended in similar cases to determine whether a direct viral infection is responsible for the clinical presentations or immune system response.

## Conclusions

Encephalopathy and encephalitis may be a presentation of COVID-19. Clinicians and health care providers should consider the presence of COVID-19 in patients with bizarre behavior even in non-severe forms of the disease during this pandemic.

## Data Availability

Not applicable.
